# In vitro Evaluation of the Potent Antileishmanial Activity of *Ferula tabasensis* Alone or in Combination with Shark Cartilage Extract Against the Standard Iranian Strain of *Leishmania major* (MRHO/IR/75/ER)

**DOI:** 10.5812/ijpr-136173

**Published:** 2023-07-06

**Authors:** Shaylin Saraei, Narges Soozangar, Mansour Miran, Fatemeh Ghaffarifar, Behnam Mohammadi-Ghalehbin, Soheila Molaei, Shabnam Asfaram

**Affiliations:** 1 Students Research Committee, School of Medicine, Ardabil University of Medical Sciences, Ardabil, Iran; 2 Zoonoses Research Center, Ardabil University of Medical Sciences, Ardabil, Iran; 3 Department of Pharmacognosy and Biotechnology, School of Pharmacy, Ardabil University of Medical Sciences, Ardabil, Iran; 4 Department of Parasitology, Faculty of Medical Sciences, Tarbiat Modares University, Tehran, Iran

**Keywords:** Ethyl Acetate Extract, Methanol Extract, Fraction, *Ferula tabasensis*, Shark Cartilage Extract, *Leishmania major*, In vitro

## Abstract

**Background:**

The available drugs for the treatment of leishmaniasis are highly toxic and extremely expensive, with low efficiency; therefore, the development of effective therapeutic compounds is essential.

**Objectives:**

The present study aimed to explore the antileishmanial effects of ethyl acetate extract, methanol extract, and fractions 1-4 (F1-F4) of *Ferula tabasensis*, alone or in combination with shark cartilage extract (ShCE), on *L. major* in vitro.

**Methods:**

In this study, ethyl acetate, methanol, and n-hexane extracts were extracted from the aerial roots of *F. tabasensis* by the maceration method. The silica gel column chromatography was used to separate n-hexane extracts at varying polarities (F1-F4 fractions). Subsequently, the effects of extracts and fractions against promastigotes were assessed by the parasite counting method microscopic inhibition test and MTT assay. Besides, their effects on the infected macrophage cells and the number of amastigotes were investigated. Cytotoxicity was evaluated in non-infected J774A.1 macrophage cells. Finally, apoptosis induction of promastigotes, including infected and non-infected macrophages, was evaluated.

**Results:**

The results indicated the highly potent activity of *F. tabasensis* extracts and F1-F4 fractions, alone or in combination with ShCE, against *L. major* promastigotes and amastigotes in a dose-dependent manner (P < 0.05). The F1 fraction and methanol extract showed markedly higher toxicity compared to the other extracts and fractions, with 50% inhibitory concentrations (IC50/72h) of 2.4 ± 0.29 and 2.9 ± 0.55 µg/mL against promastigotes and 1.79 ± 0.27 µg/mL and 1.39 ± 0.27 µg/mL against amastigotes (P < 0.001). Moreover, they had a high selectivity index (SI) due to the low toxicity of macrophages (P < 0.0001). The results of flow cytometry indicated that the percentages of apoptotic promastigote cells in contact with IC50 concentrations of F1 and methanol extract alone after 72 h were 43.83 and 43.93%, as well as 78.4%, and 65.45% for their combination with ShCE, respectively.

Also, apoptosis of infected macrophages induced by F1 and methanol extracts was estimated at 68.5% and 83.7%, respectively.

**Conclusions:**

In this study, the F1 fraction and methanol extract of *F. tabasensis* showed potent efficacy against *L. major*, associated with low toxicity and apoptosis induction. Therefore, they can be promising therapeutic candidates in future animal and even human studies.

## 1. Background

*Leishmania* species, as obligate intracellular flagellate parasites, are responsible for leishmaniasis, which is an infectious disease affecting humans and animals (1). Leishmaniasis is recognized as an important vector-borne zoonotic disease, which commonly manifests as visceral leishmaniasis (VL), cutaneous leishmaniasis (CL), mucocutaneous leishmaniasis, and diffuse cutaneous leishmaniasis (2). Nevertheless, limited effort has been made to control and prevent CL due to challenges, such as vector or main reservoir control. Among these challenges, the treatment of CL is the most important one (3). Different types of synthetic drugs are used for CL treatment, the majority of which have side effects and unsatisfactory therapeutic effects (4). Therefore, researchers are seeking more effective drugs to replace the current ones for the treatment of CL. Several natural compounds, including alkaloids, naphthoquinones, neolignans, chalcones, triterpenoids, and lignans, have been reported to exert inhibitory effects against *Leishmania* species (5).

The genus *Ferula*, which belongs to the Apiaceae family, is widely distributed throughout Southwest and Central Asia (e.g., Iran and Afghanistan), North India, and the Far East (6). Sesquiterpenes, coumarin, sesquiterpene lactones, monoterpene coumarins, prenylated coumarins, carbohydrates, flavonoids, phytoestrogen, and sulfur-containing derivatives are the main chemical compounds isolated from *Ferula* species (7). Some metabolites and extracts from some *Ferula* species have been identified to have pharmaceutical and biological activities, such as antifungal (8), antibacterial (9, 10), and antiviral (11) effects. Several studies have shown that *Ferula* species have potent antiprotozoal effects against *Leishmania major* (12), *Leishmania tropica* (13), *Plasmodium falciparum* (14), *Trichomonas vaginalis* (15), and *Echinococcus granulosus* (16).

In this study, considering the biological and pharmacological properties of shark cartilage extract (ShCE), such as anti-tumor, anti-angiogenesis, antioxidant, anti-inflammatory, immunomodulatory (17, 18), and leishmanicidal (19-21) effects, it was used alongside *Ferula tabasensis* to evaluate the synergistic or non-synergistic effects. 

## 2. Objectives

The present study mainly aimed to evaluate the leishmanicidal activity, cytotoxicity, and apoptotic affinity of ethyl acetate extract, methanol extract, and F1-F4 fractions of *F. tabasensis*, used alone or in combination with ShCE against *L. major* in vitro.

## 3. Methods

### 3.1. Plant Samples

The roots of *F. tabasensis* were collected from Tehran, Iran, in July 2020. A voucher specimen (ARD-R1) was deposited in the herbarium of the School of Pharmacy of Ardabil University of Medical Sciences, Ardabil, Iran.

### 3.2. Isolation of Extracts and Fractions

The aerial roots of *F. tabasensis* (1.5 kg) were powdered and extracted with n-hexane (2×10 L), ethyl acetate (2 × 10 L), and methanol (2×10 L) by the maceration method at room temperature, respectively. The n-hexane extract was concentrated in a rotary evaporator to obtain 45 g of a dark sticky residue. The residue was then fractionated by silica gel column chromatography (230-400 mesh size, 400 g) with an n-hexane/EtOAc gradient (100:0 to 0:100) as eluent, followed by increasing concentrations of methanol (up to 20%) in ethyl acetate. Finally, F1-F4 fractions with different polarities were obtained (22). The extracts and fractions were dissolved in 1% methanol to prepare a stock solution. It is worth mentioning that previous studies have attributed no toxicity to methanol concentrations up to 1% against promastigotes or amastigotes (23).

### 3.3. Cultivation of L. major

The standard Iranian strain of *L. major* (MRHO/IR/75/ER) was prepared by Dr. Hajjaran from the School of Public Health of Tehran University of Medical Sciences (Tehran, Iran). The promastigotes were cultured and sub-cultured in the NNN and RPMI-1640 media, which were respectively enriched with 20% fetal bovine serum (FBS) inactivated at 56ºC for 30 minutes and 1% penicillin/streptomycin (Pen-Strep) at a temperature of 24°C ± 2.

### 3.4. J774A.1 Macrophage Cell Culture

The murine macrophage cell line, J774A.1, which was purchased from the Pasteur Institute of Iran, was cultured in RPMI-1640 medium, supplemented with 12% FBS and 1% Pen-Strep at 37°C under humidified conditions in a 5% CO_2_ atmosphere. The confluent cells were obtained by adding fresh media to the flasks daily (21, 24).

### 3.5. Preparation of ShCE

The purchased shark cartilage (Bushehr Port, Persian Gulf, Iran) was used to prepare ShCE, according to the method proposed by Hassan et al., as described in our previous studies. In brief, 10 g of cartilage powder (the cartilage was cleaned, cut into small pieces, lyophilized, and powdered) was poured into 100 mL of phosphate-buffered saline (0.1 M PBS) containing guanidine hydrochloride (4 M) and phenylmethylsulfonyl fluoride (1 mM PMSF, pH=5.8) as a protease inhibitor and incubated at 2 - 8°C for 48 hours on slight stirring. Subsequently, the solution was centrifuged at 100,000 g for 45 minutes. The supernatant was then precipitated in 20% polyethylene glycol and dialyzed against PBS. The sodium dodecyl sulfate-polyacrylamide gel electrophoresis (SDS-PAGE) method was also used to evaluate the purity of proteins and represent their molecular weight against the standard protein ladder (19, 25).

### 3.6. Promastigote Assay

#### 3.6.1. Parasite Counting Method

First, the microscopic parasite counting method was used to investigate the effects of various concentrations of ethyl acetate and methanol extracts and F1-F4 fractions of *F. tabasensis* on the promastigotes of *L. major*. For this purpose, 100 µL of enriched RPMI-1640 medium, containing 1 × 10^6^ promastigotes/mL in the logarithmic growth phase, was cultured in 96-well culture plates (NuncTM) in the presence of various concentrations (1.56 - 200 µg/mL) of extracts and fractions via 24, 48, and 72 hours of incubation at 24°C. Moreover, the effects of extracts and fractions at half maximal inhibitory concentrations (IC_50_), combined with 200 µg/mL of ShCE, were assessed after 24, 48, and 72 hours. The final concentrations (e.g., 200 + 2.4 and 200 + 2.9 µg/mL) were prepared for combined drug formulations right before the test. Afterward, the average number of promastigotes was directly counted under a light microscope at 10× magnification. Amphotericin B (AmB, Gilead UK), prepared in sterile PBS based on the manufacturer’s instructions right before the experiments, and glucantime (GLU, 1.5 g/5 mL ampules; Sanofi-Aventis, France) were used as positive controls at different concentrations (1.56 - 200 µg/mL). The three wells of each plate without any drugs were considered as the negative controls (Ctrl-). The experiments were performed in triplicate (19, 26).

#### 3.6.2. MTT Assay for Promastigote Viability

The 3-(4, 5-dimethylthiazol-2-yl)-2,5-diphenyltetrazolium bromide (MTT) colorimetric assay was used to evaluate the IC_50_ concentrations. For this purpose, after seeding 100 µL of adjusted RPMI-1640 medium, containing 1×10^6^/mL logarithmic-phase promastigotes, into each of the 96-well ELISA plates, the parasites were exposed to increasing concentrations (1.56 - 200 µg/mL) of ethyl acetate and methanol extracts of *F. tabasensis*, as well as F1-F4 fractions. Also, 200 µg/mL of ShCE was used in combination with the IC_50_ concentrations of the extracts and fractions, as described earlier.

After 72 hours of incubation at 25 ± 1°C, the plates were centrifuged at 3000 rpm for 10 minutes, and the supernatant was removed and replaced with a fresh medium of the same volume. Next, 20 µL of MTT solution (5 mg MTT powder/mL in PBS) was added to each well and incubated for five hours at 37°C in a dark room. Next, the cells were centrifuged again at 3000 rpm for 10 minutes, and 100 µL of dimethylsulfoxide (DMSO) was added to the pellets. After 10 minutes, the optical densities (ODs) were measured at a wavelength of 570 nm using an ELISA reader. The viability percentage of promastigotes was determined based on the following formula:

Viability percentage = 100 × (Absorbance of treated cells - Absorbance of the blank/Absorbance of control cells - Absorbance of the blank)

Meanwhile, the promastigotes suspended in PBS with no drugs and medium with no promastigotes and drugs were respectively used as the negative control (Ctrl-) and blank (19, 27, 28).

#### 3.6.3. Intracellular Amastigote Assay

The effects of *F. tabasensis* extracts and fractions on infected macrophage cells and intra-amastigotes were evaluated, as previously described (19). Briefly, 3×10^5^ of J774A.1 macrophage (200 µL) was added to each well of 12-well plates, with small round glass coverslips at the bottom of the plate, and incubated for 12 hours at 37°C in a 5% CO_2_ atmosphere for adherence. Next, the suspended macrophages were removed by washing them with sterile PBS. To infect the macrophages, 3 × 10^6^ stationary-phase promastigotes of *L. major* (1:10 ratio) were added to the adherent macrophages and incubated again for 12 hours at 37°C in a 5% CO_2_ atmosphere with 95% relative humidity.

Free parasites were removed by washing with fresh RPMI-1640 medium after 12 hours. The infected macrophages were incubated in the presence of the extracts and fractions at different concentrations (1.56 - 200 µg/mL). After 72 hours, the slides were washed with PBS, fixed with methanol, and stained with Giemsa stain to count the infected macrophages and amastigotes under an optical microscope. The GLU and AmB were used as the positive controls at the same concentrations described in the promastigote assay. The number of amastigotes in 100 macrophage cells was counted, and the IC_50_ was determined (19, 23).

#### 3.6.4. Macrophage Cytotoxicity Assay

Since macrophage cells are the principal resident cells for *Leishmania* parasites, the J774A.1 macrophage was used in this study to assess the toxicity of ethyl acetate and methanol extracts and F1-F4 fractions of *F. tabasensis* at concentrations of 1.56 - 200 µg/mL, alone or in combination with 200 µg/mL of ShCE. For this purpose, 2 × 10^5^ cells/well were cultured in 96-well microplates with RPMI-1640 medium, containing 1% Pen-Strep and 12% FBS, and the cells were allowed to attach at a temperature of 37°C in a 5% CO_2_ atmosphere for 12 hours. Next, 100 µL of each concentration was added to the wells and incubated for another 72 hours. Finally, cytotoxicity was determined using the MTT assay, as described in the section above. Additionally, 50% cytotoxicity concentrations (CC_50_) of the extracts and fractions were determined as a 50% reduction in the cell viability of treated cells relative to untreated cells. The ratio of CC_50_ for macrophage cells to IC_50_ for amastigotes was defined as the selectivity index (SI) (13, 19).

### 3.7. Flow Cytometry

The flow cytometry method was utilized to indicate apoptotic and necrotic cells using Annexin V Apoptosis Detection Kit (MabTag, Germany), according to the manufacturer’s instructions. In brief, 1 × 10^6^ logarithmic-phase promastigotes, 1 × 10^5^ uninfected macrophages, and 200 µL of infected macrophage cells, exposed to the IC_50_ concentrations of extracts and fractions, were collected. They were first washed with a cold, sterile PBS solution and then centrifuged for 15 minutes at 1000 g. Next, 5 µL of annexin V and propidium iodide (PI), as well as 400 µL of binding buffer, were added to the cell pellets. The promastigotes and macrophage cells were incubated at 25°C and 37°C, respectively, in a dark room for 15 minutes. The test results were read in a CyFlow Space Flow cytometer (Sysmex-Partec, USA), and the collected data were analyzed in FlowJoTM Version 10.5.3 (Vancouver, BC, Canada). All the experiments were performed in triplicate, and the percentages of apoptosis and necrosis were assessed for each tested sample.

### 3.8. Statistical Analysis

Data are presented as mean and standard deviation (SD) of experiments run in triplicate. The IC_50_ values of promastigotes and amastigotes, as well as the CC_50_ values of J774A.1 macrophage cell were calculated based on the mean viability percentage of promastigotes, amastigotes, and macrophage cells, respectively, against the untreated controls. Microsoft Excel Version 16 and SPSS Version 24 (SPSS Inc., Chicago, IL, USA) was used for evaluating the results, drawing figures, and analyzing the data. Moreover, the repeated measures ANOVA test was used to analyze the results of the parasite counting method. A one-way ANOVA test, followed by Tukey's post-hoc test, was also performed to compare the IC_50_ and CC_50_ values of compounds. Finally, the SI value was calculated by determining the ratio of CC_50_ to IC_50_ for amastigotes.

## 4. Results

### 4.1. Parasite Counting Method

According to the microscopic analysis, the antileishmanial activity of *F. tabasensis* extracts and fractions against *L. major* promastigotes varied in a dose- and time-dependent manner. The number of promastigotes was markedly reduced by increasing the concentrations of extracts and fractions and also over time (from 24 to 72 hours) compared to the negative control (CTRL-) (P < 0.05). The F1 and methanol extracts were more effective against promastigotes compared to the ethyl acetate extract and other fractions (Table 1). Overall, the present results highlighted the remarkable effects of ShCE at a concentration of 200 µg/mL in combination with the IC_50_ concentrations of *F. tabasensis* extracts and fractions against the promastigotes (P < 0.05) (Table 2).

**Table 1.** The Average Number of *L. major* Promastigotes (×10^4^) in Exposure to Different Concentrations of *F. tabasensis* Extracts and Fractions After 24, 48, and 72 Hours (n = 3) ^a^

Variables	Concentration (µg/mL)								
	200	100	50	25	12.5	6.26	3.12	1.56	Control -
**Ethyl acetate**									
24	23.2 ± 2.8	18.3 ± 1.5	17.1 ± 1.7	26.7 ± 5.4	35.3 ± 3.2	30.2 ± 2.8	46.5 ± 4.5	50 ± 7.3	91.3 ± 1.5
48	21.7 ± 3.5	16 ± 4.1	13.5 ± 2.7	23 ± 2.1	31 ± 3.9	29.3 ± 3.5	42.6 ± 2.9	45.4 ± 3.2	97.5 ± 2.6
72	17.2 ± 5.3	14 ± 3.6	11.3 ± 5.4	18.7 ± 4.5	25.3 ± 3.9	27 ± 3.7	40 ± 2.5	42 ± 6.7	102.6 ± 38
**Methanol**									
24	3.6 ± 3.1	7.3 ± 5.1	10.6 ± 2.3	11.3 ± 5.1	24.3 ± 1.1	28 ± 2.1	31.6 ± 6.1	33 ± 7.2	
48	2.3 ± 4.5	2.3 ± 6.6	8.3 ± 7.1	9 ± 1.2	17.3 ± 3	24.6 ± 2.5	29 ± 6.2	30.6 ± 4.5	
72	0 ± 1.1	1.6 ± 0.8	3.4 ± 1.2	5.1 ± 3.2	14.2 ± 3.5	22.6 ± 1.5	26.5 ± 2.9	29 ± 2.1	
**F1**									
24	3 ± 1.1	3 ± 1.5	9.4 ± 2.3	12.3 ± 6.1	22.7 ± 1.4	40.3 ± 1.9	51.7 ± 3.1	65.3 ± 6.3	
48	1.7 ± 1.6	2 ± 2.1	7 ± 4.5	10 ± 5.1	22 ± 2.1	36 ± 1. 7	51.6 ± 3.1	61.5 ± 5.9	
72	0 ± 0.85	0.5 ± 1.9	3.3 ± 2.4	9.5 ± 1.6	19 ± 4.1	32.7 ± 2.7	49.6 ± 3.1	60.7 ± 5.1	
**F2**									
24	3.3 ± 1.4	5.2 ± 1.9	10.3 ± 3.1	24.7 ± 1.8	35.3 ± 5.2	54.3 ± 3.5	64 ± 1.6	74 ± 3.3	
48	2 ± 1.1	5 ± 1.1	9.1 ± 1.13	20.3 ± 1.1	34.7 ± 1.1	45.2 ± 1.1	58 ± 1.1	72.3 ± 1.1	
72	0.2.1	4 ± 4.1	8.6 ± 1. 7	18 ± 3.2	31.3 ± 3.3	42.7 ± 3.4	56 ± 0.9	70.6 ± 1. 7	
**F3**									
24	2 ± 1.8	5.3 ± 1.3	12.2 ± 1.7	35.5 ± 4.1	49 ± 2.1	64.3 ± 2.2	72.3 ± 1.6	75.7 ± 1.3	
48	1.3 ± 3.3	4 ± 2.1	11.3 ± 4.3	31.2 ± 1.7	45 ± 5.1	60 ± 4.5	69 ± 1. 3	73.8 ± 6.1	
72	0 ± 1.7	3.2 ± 1.3	10 ± 6.1	30.4 ± 2. 3	42.7 ± 7.1	58.4 ± 2.1	61.4 ± 3.1	67.3 ± 5.1	
**F4**									
24	17 ± 1.4	29 ± 3.7	36.3 ± 1.3	45 ± 3.1	52.6 ± 1. 7	60.6 ± 1.4	68 ± 2.5	75.3 ± 4.3	
48	16.2 ± 4.6	27 ± 1.9	33.3 ± 1.2	41.4 ± 4.1	45 ± 5.1	54.2 ± 2.5	61.1 ± 3.1	72.1 ± 4.5	
72	15 ± 1.7	25 ± 2.6	31 ± 3.1	38 ± 1.3	40.5 ± 1.9	48 ± 3.4	56 ± 2.2	71.8 ± 7.1	
**AmB**									
24	14 ± 3.1	21 ± 2.5	27 ± 3.6	37 ± 2.1	55 ± 1.8	58 ± 2.4	62 ± .5	65 ± 0.8	
48	10 ± 1.1	16 ± 1.6	21 ± 2.5	29 ± 2.2	51 ± 2.7	55 ± 1.9	57 ± 1.2	60 ± 1.1	
72	5 ± 1.3	11 ± 4.1	17 ± 1.8	22 ± 2.5	45 ± 4.1	50 ± 2.8	52 ± 3.5	57.3 ± 2.1	
**GLU**									
24	83 ± 1.5	84 ± 1.5	85 ± 1.1	86 ± 1.2	87 ± 1.1	88 ± 2.5	90 ± .2	91 ± 1.8	
48	75 ± 1.5	80 ± 1.8	84 ± 2.1	87 ± 2.5	92 ± 2.1	94 ± 1.2	96 ± 1.5	97 ± 1.5	
72	50 ± 1.9	55 ± 2.1	57 ± 1.5	59 ± 2.3	62 ± 1.1	67 ± 2.5	69 ± 1.5	71 ± 2.9	

^a^ Data are expressed as mean ± SD.

**Table 2.** The Effects of *F. tabasensis* Extracts and Fractions at IC_50_ Concentrations Combined with 200 µg/mL of ShCE on the Growth of *L. major* Promastigotes (×10^4^) After 24, 48, and 72 Hours (n = 3) ^a^

Variables	Concentration (µg/mL)	
	ShCE (200 µg/mL)	Control -
**ShCE (200 µg/mL)**		
24	48 ± 2.5	91.3 ± 1.5
48	42 ± 2.3	97.5 ± 2.6
72	35 ± 1.8	102.6 ± 3.8
**Ethyl acetate (3.8 µg/mL)**		
24	27.8 ± 2.3	
48	24.5 ± 1.5	
72	11.2 ± 2.1	
**Methanol (2.9 µg/mL)**		
24	17 ± 3.3	
48	12 ± 1.5	
72	9 ± 2.6	
**F1 (2.4 µg/mL)**		
24	21 ± 2.6	
48	16 ± 2.3	
72	12 ± 1.5	
**F2 (4.85 µg/mL)**		
24	37.5 ± 3.2	
48	30 ± 1.3	
72	22.8 ± 1.9	
**F3 (4.2785 µg/mL)**		
24	33.8 ± 3.8	
48	25.5 ± 2.3	
72	16.7 ± 1.2	
**F4 (33.5 µg/mL)**		
24	42 ± 1.6	
48	36.9 ± 2.2	
72	32 ± 1.9	
**AmB (33.9 µg/mL)**		
24	23 ± 2.1	
48	17 ± 1.5	
72	12 ± 3.7	
**GLU (420 µg/mL)**		
24	73 ± 1.9	
48	65 ± 1.3	
72	43 ± 1.4	

^a^ Data are expressed as mean ± SD.

### 4.2. MTT Assay for Promastigote Viability

Based on the results, the viability of promastigotes against various concentrations of extracts and fractions (1.56 - 200 µg/mL) decreased in a dose-dependent manner and efficacy increased with an enhancement of drug concentrations. In other words, concentrations of 1.56 and 200 µg/mL resulted in the highest and lowest viability percent, respectively (P < 0.001).

**Figure 1.**
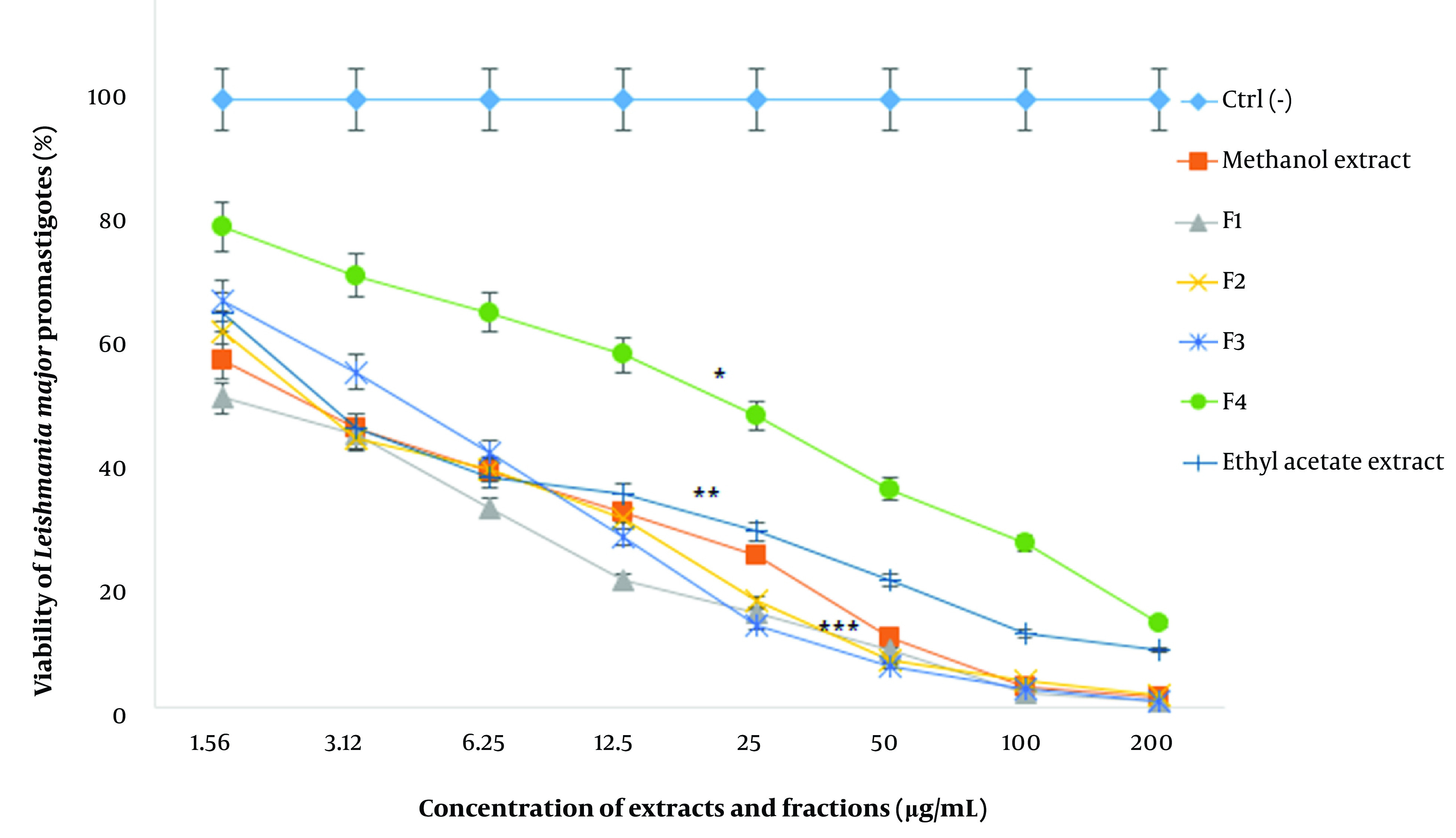
The viability percentage of *L. major* promastigotes against different concentrations (µg/mL) of *F. tabasensis* extracts and fractions after 72 hours. Data are shown as mean ± SD of triplicate experiments. The results are analyzed by Dunnett’s test (*P < 0.05, **P < 0.01, *** P < 0.001, compared to the control group (Ctrl-)).

The IC_50_ values of ethyl acetate extract, methanol extract, and F1-F4 fractions were 3.8 ± 1.1, 2.9 ± 0.5, 2.4 ± 0.2, 4.8 ± 1.2, and 4.2 ± 1.8, and 33.5 ± 2.6 µg/mL, respectively (Figure 1). The highest and lowest IC_50_ values were attributed to the F4 and F1 fractions, respectively (P < 0.001). Also, the IC50 values of AmB and GLU, as the positive controls, were measured to be 33.9 ± 5.1 and 420 ± 1.9 µg/mL for the promastigotes, which were significantly higher than the other extracts and fractions (P < 0.001).

The results of statistical analysis indicated significant differences between all the groups in comparison to the control group (P < 0.05).

The results of statistical analysis indicated significant differences between all the groups compared to the control group (P < 0.05).

### 4.3. Amastigote Assay

The results of the amastigote assay revealed that different concentrations of *F. tabasensis* extracts and fractions resulted in a remarkable reduction in both the number of infected macrophages and the number of intra-amastigotes in comparison to the negative control (Ctrl-) (P < 0.05). Overall, 42.5% of J774A.1 macrophage cell was infected in the Ctrl- group. Concerning the effects of exposure to the extracts and fractions, it was found that the ethyl acetate and methanol extracts, F1 fraction, and F3 fraction significantly decreased the number of infected macrophage cells, as well as the mean number of intracellular amastigotes at different concentrations compared to the other studied compounds and positive controls (P < 0.001) (Table 3). Interestingly, the IC_50_ was lower than the IC_50_ calculated for the promastigotes, except for the F2 fraction (Table 4). On the other hand, ShCE at 200 µg/mL, combined with various concentrations of extracts or fractions, significantly reduced the number of infected macrophages, as well as the mean number of intra-amastigotes (P < 0.001); therefore, its combined use with *F. tabasensis* extracts and fractions was more efficient than its independent use (data are not shown).

**Table 3.** Effects of Different Concentrations of Ethyl Acetate and Methanol Extracts and F1-F4 Fractions of *F. tabasensis* on the Infected Macrophages and Intracellular Amastigotes After 72 Hours (n = 3)

Variables	Concentration (µg/mL)								
	200	100	50	25	12.5	6.26	3.12	1.56	Control -
**Ethyl acetate**									
Infected Mϕ (%)	15 ± 1.4	19 ± 2.8	24.8 ± 3.2	33 ± 2.1	37.5 ± 2.4	38 ± 2.7	39 ± 3.1	40.4 ± 1.3	42.5 ± 2.1
Amastigotes/macrophages	0.08 ± 0.01	0.11 ± 0.03	0.29 ± 0.07	0.62 ± 0.06	1.1 ± 0.05	1.98 ± 0.1	2.29 ± 0.5	3.29 ± 0.5	5.33 ± 0.33
**Methanol**									
Infected Mϕ (%)	13 ± 2.4	17 ± 1.9	25. ± 1.1	31 ± 0.9	35 ± 1.1	39 ± 2.5	40 ± 0.4	40 ± 1.2	
Amastigotes/macrophage	0.02 ± 0.03	0.05 ± 0.03	0.13 ± 0.03	0.48 ± 0.05	0.96 ± 0.06	1.66 ± 0.05	2.11 ± 0.08	2.35 ± 0.1	
**F1**									
Infected Mϕ (%)	10 ± 2.5	15 ± 1.2	25 ± 1.6	30 ± 0.5	35 ± 1.1	39 ± 2.9	40 ± 0.4	40 ± 1.7	
Amastigotes/macrophages	0.05 ± 0.03	0.09 ± 0.01	0.21 ± 0.96	0.85 ± 0.05	1.12 ± 0.07	1..85 ± 0.09	2.1 ± 0.05	2.85 ± 0.08	
**F2**									
Infected Mϕ (%)	21 ± 2.1	25 ± 0.2	31 ± 3.6	35 ± 2.5	39 ± 1.1	39 ± 1.6	39 ± 1.7	40 ± 1.3	
Amastigotes/macrophages	0.92 ± 0.12	0.96 ± 0.07	1.15 ± 0.17	1.7 ± 0.05	2.1 ± 0.15	2.38 ± 0.11	3.2 ± 0.21	4.5 ± 0.1	
**F3**									
Infected Mϕ (%)	17 ± 2.1	21 ± 1.2	25 ± 3.1	33 ± 3.5	35 ± 1.8	38 ± 2.6	39 ± 1.4	40 ± 1.3	
Amastigotes/ macrophage	0.23 ± 0.05	0.85 ± 0.07	0.96 ± 0.01	1 ± 0.03	1.1 ± 0.09	1.5 ± 0.23	1.89 ± 0.05	2.95 ± 0.1	
**F4**									
Infected Mϕ (%)	26 ± 1.1	28 ± 2.7	31 ± 3.1	36 ± 2.8	37 ± 4.2	39 ± 0.7	40 ± 0.2	40 ± 1.1	
Amastigotes/macrophages	0.99	1.1	1.55	2.79	3.26	3.95	4.26	5.25	
**AmB**									
Infected Mϕ (%)	28 ± 0.2	31 ± 1.1	34 ± 0.8	36 ± 0.5	39 ± 0.2	39 ± 1.6	40 ± 1.2	40 ± 1.3	
Amastigotes/macrophages	1.1 ± 0.21	1.85 ± 0.15	2.58 ± 0.31	3.2 ± 0.29	3.8 ± 0.52	4 ± 0.18	4.3 ± 0.21	4.9 ± 0.11	
**GLU**									
Infected Mϕ (%)	25 ± 1.5	31 ± 2.5	34 ± 1.5	35 ± 1.2	38 ± 1.5	41 ± 0.5	42 ± 0.5	42 ± 1.3	
Amastigotes/macrophages	2.75 ± 0.14	3.1 ± 0.08	3.9 ± 0.23	4.5 ± 0.11	4.7 ± 0.09	4.9 ± 0.1	5 ± 0.02	5.1 ± 0.11	

Abbreviation: Mφ, macrophages.

^a^ Data are expressed as mean ± SD.

**Table 4.** Comparison of IC_50_ Values of Ethyl Acetate and Methanol Extracts and F1-F4 Fractions of *F. tabasensis* for Promastigotes, IC_50_ Values for Amastigotes, and CC_50_ Values for the J774A.1 Macrophage Cells

Extracts and Fractions	Promastigotes, IC_50_ ± SD (µg/mL)	Amastigotes, IC_50_ ± SD (µg/mL)	J774 Macrophage cells, CC_50_ ± SD (µg/mL)	SI=CC_50_/IC_50_ of Amastigotes
**Ethyl acetate**	3.8 ± 1.13	2.95 ± 0.26	159.9 ± 4.2	53.9
**Methanol**	2.9 ± 0.55	1.39 ± 0.68	76 ± 7.9	54.7
**F1**	2.4 ± 0.29	1.79 ± 0.27	123.9 ± 1.8	69.2
**F2**	4.85 ± 1.2	5.92 ± 0.08	19.5 ± 2.6	1.6
**F3**	4.27 ± 1.82	2.25 ± 0.58	132.8 ± 3.1	59
**F4**	33.5 ± 2.66	28.9 ± 0.25	295 ± 15.2	10.2
**GLU**	420 ± 1.9	210 ± 2.3	845 ± 10.5	4.02
**AmB**	33.9 ± 5.1	55.6 ± 0.21	315 ± 9.8	5.7

Abbreviations: IC_50_, 50% inhibitory concentration; CC_50_, 50% cytotoxicity concentration, SI: ratio of toxicity toward macrophage cells to toxicity toward amastigote cells.

The results of statistical analysis revealed significant differences between all groups compared to the control group (P < 0.05).

### 4.4. Cytotoxicity of Macrophages

Similar to promastigotes and amastigotes, the cytotoxicity of macrophages was based on the concentrations of extracts and fractions (P < 0.05). Evaluation of all extracts and fractions, except F2, indicated the low toxic effects of macrophages (SI>10). As shown in Table 4, the highest and lowest IC_50_ values were 295 ± 15.2 and 19.5 ± 2.6 µg/mL, attributed to the F4 and F2 fractions, respectively (Table 4).

### 4.5. Flow Cytometry

The exposure of stationary-phase *L. major* promastigotes, as well as infected and non-infected macrophages, to the extracts and fractions of *F. tabasensis* at their IC_50_ concentrations after 72 hours induced early apoptosis (Q3, positive for Annexin V), late apoptosis (Q2, positive for Annexin V and PI), necrosis (Q1, positive for PI), and alive cells (Q4, negative for Annexin V and PI) compared to the untreated control cells. The percentage of apoptotic and necrotic cells varied depending on the extract or fraction.

**Figure 2.**
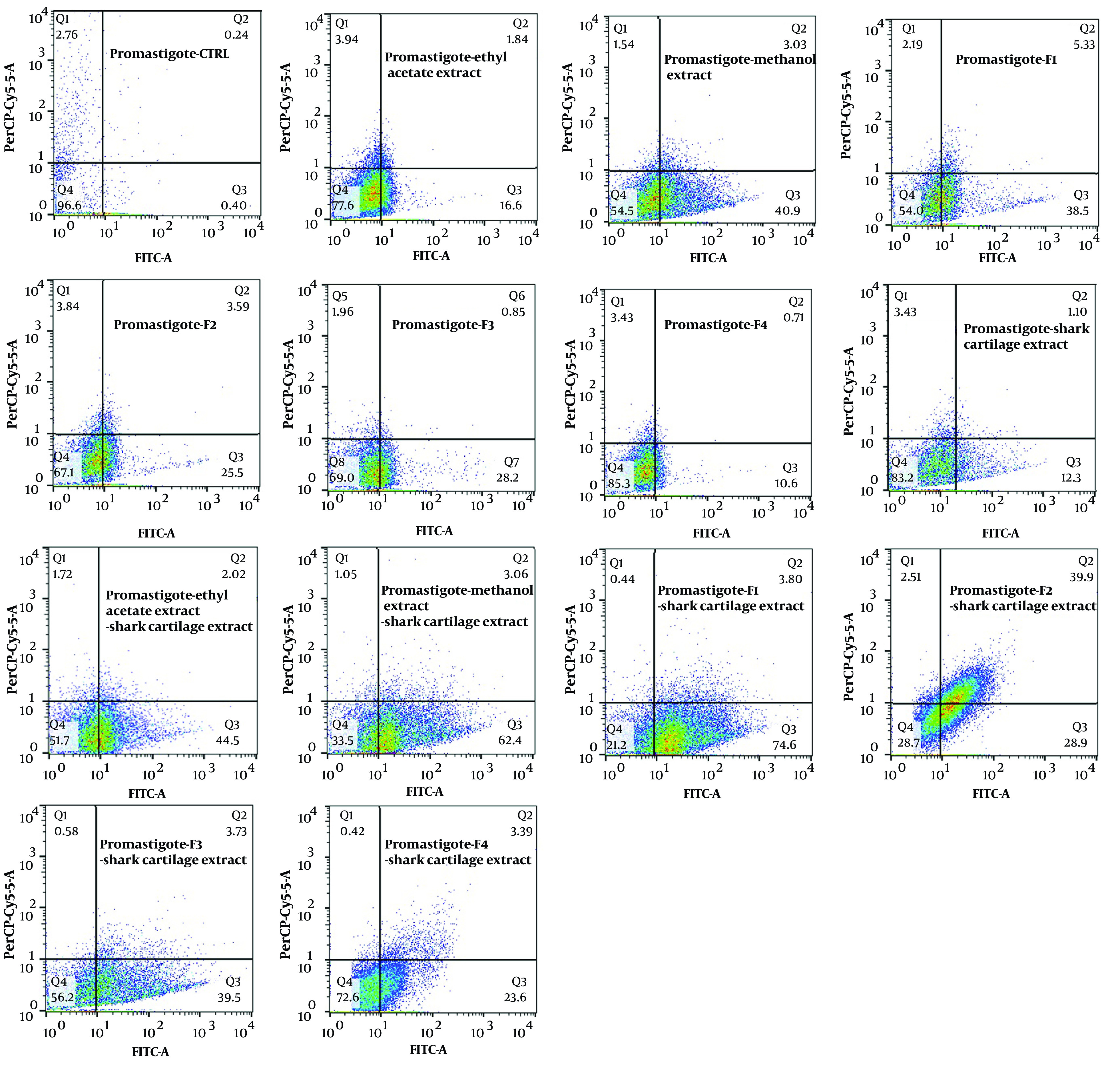
The apoptotic and necrotic profiles of *L. major* promastigotes in exposure to IC_50_ concentrations of ethyl acetate and methanol extracts and F1-F4 fractions of *F. tabasensis* used alone or in combination with ShCE

**Figure 3.**
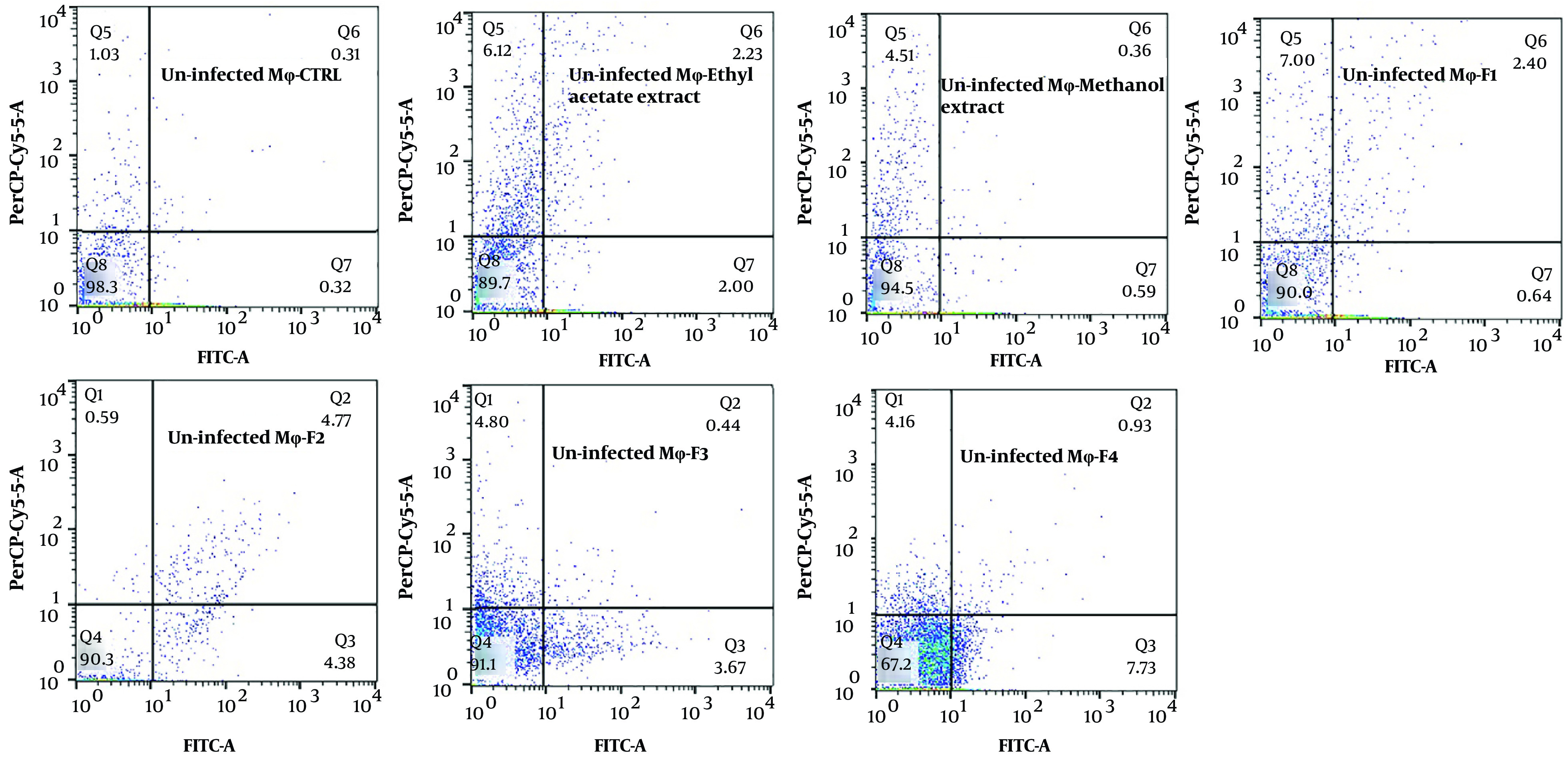
The apoptotic and necrotic profiles of *L. major* promastigotes in exposure to IC_50_ concentrations of ethyl acetate and methanol extracts and F1-F4 fractions of *F. tabasensis* used alone or in combination with non-infected macrophages

**Figure 4.**
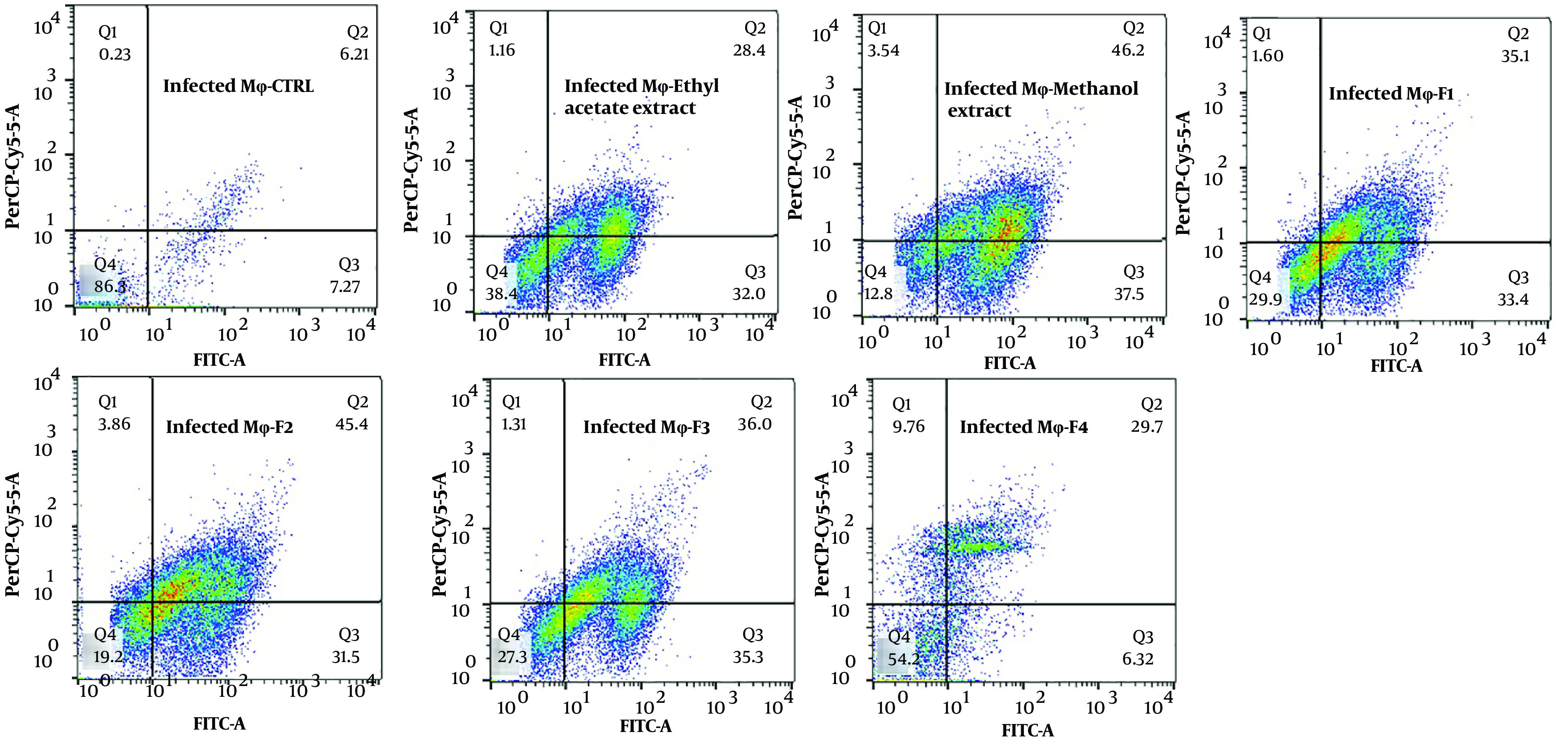
The apoptotic and necrotic profiles of *L. major* promastigotes in exposure to IC_50_ concentrations of ethyl acetate and methanol extracts and F1-F4 fractions of *F. tabasensis* used alone or in combination with infected macrophage cells

As shown in Figure 2 - 4, significant early apoptosis of promastigote cells was detected in exposure to the methanol extract and F1 fraction. Additionally, the results of flow cytometry indicated the synergistic effects of extracts and fractions when combined with ShCE. Interestingly, the percentage of apoptosis (early and late apoptosis) was higher in the infected macrophage cells compared to the promastigotes and non-infected macrophages. There were significant differences in terms of early and late apoptosis and also necrosis of all extracts and fractions only or combined with ShCE compared to the negative control (CTRL) (P < 0.001).

## 5. Discussion

The results of the present study revealed that the ethyl acetate and methanol extracts and F1-F4 fractions of *F. tabasensis* had potential activities, alone or synergistically with ShCE, against both *L. major* promastigotes and intra-macrophage amastigotes in a dose-dependent manner. The IC_50_ values for the promastigotes were 3.8 ± 1.13, 2.9 ± 0.55, 2.4 ± 0.29, 4.85 ± 1.2, 4.27 ± 1.82, and 33.5 ± 2.66 µg/mL in the ethyl acetate and methanol extracts and F1-F4 fractions, respectively. The corresponding values for GLU and AmB, as the reference drugs, were 420 ± 1.9 and 33.9 ± 5.1 µg/mL, respectively.

Since macrophage cells are the main host cells for *Leishmania* parasites, one of the most important steps in the fight against *Leishmania* parasites is to prevent the infectivity of macrophage cells and destroy them (29). The present findings showed the inhibitory effects of all extracts and fractions against intracellular amastigotes of *L. major* as the main causative agent of Old World CL. The IC_50_ value for amastigotes was lower than that of promastigotes (except F2). Based on the results, the combination of ShCE at 200 µg/mL with *Ferula* extracts and fractions at IC_50_ concentrations was more efficient than either of the drugs used alone (P < 0.001). However, the toxicity of non-infected macrophage cells was low, and the SI values were > 10 (except F2), representing a safety index for the application of these extracts and fractions to eliminate infected macrophages.

A wide range of antimicrobial (7, 13, 30-33), anthelminthic (34-38), and antiprotozoal (7, 13, 15, 33, 38-40) effects have been attributed to *Ferula* species. The antileishmanial effects of various oils, extracts, and fractions of the genus *Ferula* have been examined in previous studies (7, 13, 31, 33, 41, 42). Our findings are in agreement with the results reported by Vahdani et al., which indicated the high in vitro activity of *F. assa-foetida* ethanol extract (IC50 = 2 ± 0.12, ID50 = 0.65 ± 0.02 µg/mL) against promastigotes and amastigotes of *L. major*, respectively (41). In another study by these researchers, the aqueous extract of *F. assa-foetida* exhibited high efficacy against *L. major* promastigotes (IC_50_ = 3.6 µg/mL) (41).

Additionally, Bafghi et al. reported the significant preventive effects (> 90%) of *F. assa-foetida* (oleo-gum resin) on stationary- and logarithmic-phase *L. major*, using the slide method after 72 hours. Also, the viability of parasites significantly decreased in both growth phases using all drug concentrations compared to the control (43). Moreover, Mahmoudvand et al. found the presence of myrtenal, linalool, terpinolene, terpinen-4-ol, and β-phellandrene in the essential oil of *F. macrecolea* and reported its great antileishmanial effects in vitro (13). Besides, Andrade et al. reported the slight effects of *F. galbaniflua* essential oil on *L. amazonensis* promastigotes and brine shrimp (IC_50_ = 95.70 ± 1.82 µg/mL and CC_50_ = 377.26 ± 2.71 µg/mL, respectively) (44).

Recently, Mahmoudvand et al. observed the potential leishmanicidal effects of *F. macrecolea* essential oils and terpinolene against the promastigotes and amastigotes of *L. tropica*. The IC_50_ values of *F. macrecolea* essential oil and terpinolene against promastigotes were 27.6 and 11.6 µg/mL, respectively. However, their IC_50_ values against amastigotes were 42.3 and 19.6 µg/mL, respectively. The CC_50_ values of their compounds were also 471.3 and 207.3 µg/mL for the essential oil and terpinolene, respectively (13). Our results are consistent with their findings of both promastigote and amastigote assays. Moreover, in a study by Mohammadhosseini et al., the antileishmanial activities of three new compounds of the genus *Ferula*, including fnarthexone, fnarthexol, and conferol, were discussed, and the moderate activities of fnarthexone and fnarthexol with IC_50_ values of 43.77 ± 0.56 and 46.81 ± 0.81 µg/mL, respectively, were reported. However, the greatest antileishmanial activity, with the highest IC_50_ value, was attributed to conferol (11.51 ± 0.09 µg/mL) (45).

Generally, there are very few studies on the chemical composition of *F. tabasensis*. In a study by Bigdeli et al., the compounds of the Iranian *Ferula* genus were investigated. The bioactive and major compounds, as well as their biological activities, were variable with *Ferula* species, and volatile sesquiterpenes were the main components of *F. tabasensis* (46). In another study by Panahi et al., the chemo diversity of volatile compounds of *F. tabasensis*, along with the other five Ferula species, was determined. Overall, α-pinene, myrcene, thiophene derivatives, sabinene, nonane, octane, β-pinene, and carotol were the major constituents of some *Ferula* species, especially *F. tabasensis* (47).

The genus *Ferula* is mainly characterized by the presence of sesquiterpenes and sesquiterpene coumarins. Meanwhile, the main biological activity of the genus *Ferula* is ascribed to terpenoid compounds, including monoterpenes, such as α-pinene, β-pinene, myrcene, and limonene, and sesquiterpenes, such as β-caryophyllene, germacrene B, germacrene D, and δ-cadinene (48). It is known that sesquiterpenes and their oxygenated derivatives, alcohols, aliphatic aldehydes, and esters from volatile fractions are the main components of *F. tabasensis* (7, 46). These compounds lead to the discharge of adenosine triphosphatase and trigger mitochondrial membrane depolarization (49).

The strong antileishmanial activities of sesquiterpenes (50, 51), monoterpenes, sulfur-containing compounds (51), and volatile terpenoids from the genus *Ferula* (48) have been described in the literature. In our previous research, we found that in vitro exposure of promastigotes to ShCE has significant effects, including a reduction in the growth rate and viability of promastigotes, besides synergistic effects with artemisinin on both promastigotes and amastigotes in vitro and in vivo (21). The present results, for the first time, revealed that both extracts and fractions of *F. tabasensis*, combined with ShCE, exerted enhanced leishmanicidal effects against *L. major*.

Flow cytometry is an alternative technique for determining the type of programmed cell death, including early and late apoptosis and necrosis, and also for examining the effects of extracts or fractions on viability or mortality (52). It has been indicated that *Leishmania* prevents the apoptosis of infected macrophage cells. On the other hand, apoptosis occurs in *Leishmania* amastigotes and promastigotes following exposure to drugs and herbal extracts (53). In the present study, the results of flow cytometry confirmed the promastigote and amastigote assay results, which suggested significant apoptosis at IC_50_ concentrations of all extracts and fractions. The percentage of apoptosis (early and late) of promastigotes following exposure to ethyl acetate and methanol extracts and F1-4 fractions of *F. tabasensis* was measured to be 18.44%, 44.2%, 43.83%, 29.09%, 29.05%, and 11.3%, respectively; these values also increased when the extracts and fractions were combined with ShCE.

In this regard, Gharaei et al. reported the apoptosis-inducing effects of *F. gummosa Boiss* extracts in AGS, a human adenocarcinoma cell line. In this study, the ethanol extract from plant flowers induced high apoptosis (78%) in the promastigote cells (54). Moreover, in a study by Mousavi et al., the apoptotic effects of auraptene, as one of the key components of 7-prenyloxycoumarins from *F. szowitsiana*, were documented in the MCF-7 cell line (IC_50_ = 59.7 µM). In this study, DNA fragmentation was introduced as one of the underlying mechanisms of component-induced apoptosis (55).

### 5.1. Conclusions

Owing to the potent antileishmanial activity of *F. tabasensis* extracts and fractions against *L. major*, especially the methanol extract and F1 fraction used alone or in combination with ShCE, they can be not only introduced as new drug alternatives in antileishmanial therapy, but also support future research for the development of highly effective, affordable, and reliable medicines.

## References

[1] Kumar A (2020). Survival Strategies of Leishmania Parasite: Too Many Questions and Few Answers.. Curr Pharmacol Rep..

[2] Costa-da-Silva AC, Nascimento DO, Ferreira JRM, Guimaraes-Pinto K, Freire-de-Lima L, Morrot A (2022). Immune Responses in Leishmaniasis: An Overview.. Trop Med Infect Dis..

[3] Stockdale L, Newton R (2013). A review of preventative methods against human leishmaniasis infection.. PLoS Negl Trop Dis..

[4] Uliana SRB, Trinconi CT, Coelho AC (2018). Chemotherapy of leishmaniasis: present challenges.. Parasitology..

[5] Musa YM, Haruna AK, Ilyas M, Yaro AH, Ahmadu AA, Usman H (2007). Phytochemical, analgesic and anti-inflammatory effects of the ethylacetate extract of the leaves of Pseudocedrella kotschyii.. Afr J Tradit Complement Altern Med..

[6] Hosseinzadeh N, Shomali T, Hosseinzadeh S, Raouf Fard F, Pourmontaseri M, Fazeli M (2020). Green synthesis of gold nanoparticles by using Ferula persica Willd. gum essential oil: production, characterization and in vitro anti-cancer effects.. J Pharm Pharmacol..

[7] Sonigra P, Meena M (2020). Metabolic Profile, Bioactivities, and Variations in the Chemical Constituents of Essential Oils of the Ferula Genus (Apiaceae).. Front Pharmacol..

[8] Iranshahi M, Fata A, Emami B, Shahri BMJ, Bazzaz BSF (2008). In Vitro Antifungal Activity of Polysulfides-Rich Essential Oil of Ferula Latisecta Fruits against Human Pathogenic Dermatophytes.. Nat Prod Commun..

[9] Dastan D, Salehi P, Aliahmadi A, Gohari AR, Maroofi H, Ardalan A (2016). New coumarin derivatives from Ferula pseudalliacea with antibacterial activity.. Nat Prod Res..

[10] Rahman MU, Gul S, Odhano EA (2008). Antimicrobial activities of Ferula assafoetida oil against gram positive and gram negative bacteria.. Am Eur J Agric..

[11] Lee CL, Chiang LC, Cheng LH, Liaw CC, Abd El-Razek MH, Chang FR (2009). Influenza A (H(1)N(1)) Antiviral and Cytotoxic Agents from Ferula assa-foetida.. J Nat Prod..

[12] Iranshahi M, Arfa P, Ramezani M, Jaafari MR, Sadeghian H, Bassarello C (2007). Sesquiterpene coumarins from Ferula szowitsiana and in vitro antileishmanial activity of 7-prenyloxycoumarins against promastigotes.. Phytochemistry..

[13] Mahmoudvand H, Ghasemian Yadegari J, Khalaf AK, Hashemi MJ, Dastyarhaghighi S, Salimikia I (2022). Chemical composition, antileishmanial, and cytotoxic effects Ferula macrecolea essential oil against Leishmania tropica.. Parasite Epidemiol Control..

[14] Esmaeili S, Naghibi F, Mosaddegh M, Sahranavard S, Ghafari S, Abdullah NR (2009). Screening of antiplasmodial properties among some traditionally used Iranian plants.. J Ethnopharmacol..

[15] Khanmohammadi M, Ganji S, Reyhani Rad S (2014). Anti-protozoan Effects of Methanol Extracts of the Ferula szowitsiana on the Trichomonas Vaginalis Trophozoites in vitro.. Int J Women's Health Reprod..

[16] Alyousif MS, Al-Abodi HR, Almohammed H, Alanazi AD, Mahmoudvand H, Shalamzari MH (2021). Chemical Composition, Apoptotic Activity, and Antiparasitic Effects of Ferula macrecolea Essential Oil against Echinococcus granulosus Protoscoleces.. Molecules..

[17] Merly L, Smith SL (2017). Murine RAW 264.7 cell line as an immune target: are we missing something?. Immunopharmacol Immunotoxicol..

[18] Vasarri M, Degl'Innocenti D (2022). News and Updates from 2022 on Antioxidant and Anti-Inflammatory Properties of Marine Products.. Mar Drugs..

[19] Molaie S, Ghaffarifar F, Hasan ZM, Dalimi A (2019). Enhancement Effect of Shark Cartilage Extract on Treatment of Leishmania infantum with Artemisinin and Glucantime and Evaluation of killing Factors and Apoptosis in-vitro Condition.. Iran J Pharm Res..

[20] Ghafarifar F, Molaie S, Abazari R, Hasan ZM, Foroutan M (2020). Fe3O4@Bio-MOF Nanoparticles Combined with Artemisinin, Glucantime(R), or Shark Cartilage Extract on Iranian Strain of Leishmania major (MRHO/IR/75/ER): An In-Vitro and In-Vivo Study.. Iran J Parasitol..

[21] Ghaffarifar F, Molaei S, Mohammad Hassan Z, Dayer MS, Dalimi A, Nasiri V (2021). In Vitro and In Vivo Anti-parasitic Activity of Artemisinin Combined With Glucantime and Shark Cartilage Extract on Iranian Strain of Leishmania major (MRHO/IR/75/ER).. Jundishapur J Microbiol..

[22] Khamidulla K, Sobithon SMU, Akbarjon N, Tokxirjanovna UZ, Kizi KSA (2021). The Extraction and Fractionation of Ferula Samarcandica Root Exracts.. Universum Chem Biol..

[23] El-On J, Ozer L, Gopas J, Sneir R, Golan-Goldhirsh A (2009). Nuphar lutea: in vitro anti-leishmanial activity against Leishmania major promastigotes and amastigotes.. Phytomedicine..

[24] Benitez D, Medeiros A, Quiroga C, Comini MA (2022). A Simple Bioluminescent Assay for the Screening of Cytotoxic Molecules Against the Intracellular Form of Leishmania infantum.. Methods Mol Biol..

[25] Molaie S, Ghaffarifar F, Dalimi A, Zuhair MH, Sharifi Z (2019). Evaluation of synergistic therapeutic effect of shark cartilage extract with artemisinin and glucantime on visceral leishmaniasis in BALB/c mice.. Iran J Basic Med Sci..

[26] Tavakoli P, Ghaffarifar F, Delavari H, Shahpari N (2019). Efficacy of manganese oxide (Mn(2)O(3)) nanoparticles against Leishmania major in vitro and in vivo.. J Trace Elem Med Biol..

[27] Saini S, Bharati K, Shaha C, Mukhopadhyay CK (2017). Zinc depletion promotes apoptosis-like death in drug-sensitive and antimony-resistance Leishmania donovani.. Sci Rep..

[28] Paladi Cde S, Pimentel IA, Katz S, Cunha RL, Judice WA, Caires AC (2012). In vitro and in vivo activity of a palladacycle complex on Leishmania (Leishmania) amazonensis.. PLoS Negl Trop Dis..

[29] Lievin-Le Moal V, Loiseau PM (2016). Leishmania hijacking of the macrophage intracellular compartments.. FEBS J..

[30] Ghannadi A, Fattahian K, Shokoohinia Y, Behbahani M, Shahnoush A (2014). Anti-Viral Evaluation of Sesquiterpene Coumarins from Ferula assa-foetida against HSV-1.. Iran J Pharm Res..

[31] Zhou Y, Xin F, Zhang G, Qu H, Yang D, Han X (2017). Recent Advances on Bioactive Constituents in Ferula.. Drug Dev Res..

[32] Boghrati Z, Iranshahi M (2019). Ferula species: A rich source of antimicrobial compounds.. J Herb Med..

[33] Amin A, Tuenter E, Cos P, Maes L, Exarchou V, Apers S (2016). Antiprotozoal and Antiglycation Activities of Sesquiterpene Coumarins from Ferula narthex Exudate.. Molecules..

[34] Kareparamban JA, Nikam PH, Jadhav AP, Kadam VJ (2012). Ferula foetida" Hing": a review.. Res J Pharm Biol Chem Sci..

[35] Tavassoli M, Jalilzadeh-Amin G, Fard VRB, Esfandiarpour R (2018). The in vitro effect of Ferula asafoetida and Allium sativum extracts on Strongylus spp.. Ann Parasitol..

[36] Moudgil AD, Moudgil P, Sharma D, Daundkar PS, Agnihotri RK (2020). In vitro protoscolicidal efficacy appraisal of methanolic herbal extracts against hydatid cysts.. Vet Arh..

[37] Badar SN, Iqbal Z, Sajid MS, Rizwan HM, Shareef M, Malik MA (2021). Comparative anthelmintic efficacy of Arundo donax, Areca catechu, and Ferula assa-foetida against Haemonchus contortus.. Rev Bras Parasitol Vet..

[38] Panda SK, Daemen M, Sahoo G, Luyten W (2022). Essential Oils as Novel Anthelmintic Drug Candidates.. Molecules..

[39] Vazini H, Rahimi Esboei B (2018). In vitro study of the effect of hydroalcholic extracts of Carum copticum and Ferula asafetida against Trichomonas vaginalis.. Sci J Kurdistan Univ Med Sci..

[40] Apers S, Pieters L (2016). Phytochemical and Biological Investigations on Ferula Narthex Exudate: Phytochemical and Biological Investigations on Medicinal Plants from Pakistan..

[41] Vahdani M, Khoshzaban F (2012). In vitro antileishmanial activity of Ferula Assa-foetida Ethanol extracts aginst Leishmania major promastigotes strin MRHO/IR/75/ER.. Proceeding of National congress of Medicinal Plants..

[42] Rad MS, Khoshzaban F, Naeini A (2012). In vitro effects of Aqueous Ferula Assa-foetida extracts on Leishmania major promastigotes.. Proceeding of National congress of Medicinal Plants..

[43] Bafghi AF, Bagheri SM, Hejazian SH (2014). Antileishmanial activity of Ferula assa-foetida oleo gum resin against Leishmania major: An in vitro study.. J Ayurveda Integr Med..

[44] Andrade MA, Azevedo CD, Motta FN, Santos ML, Silva CL, Santana JM (2016). Essential oils: in vitro activity against Leishmania amazonensis, cytotoxicity and chemical composition.. BMC Complement Altern Med..

[45] Mohammadhosseini M, Venditti A, Sarker SD, Nahar L, Akbarzadeh A (2019). The genus Ferula: Ethnobotany, phytochemistry and bioactivities – A review.. Ind Crops Prod..

[46] Bigdeli M, Barazandeh MM, Bigdeli B, Sefid Kon F (2007). Identification composition of essential oils of roots, seeds and aerial parts of five wild plants of Ferula ovina, F. tabasensis, Torilis arvensis, Bupleurum lancifolium and Crambe orientalis.. Agric Res Educ Promot Organ..

[47] Panahi M, Rezaee M, Jaimand K (2020). A review of phytochemistry and phylogeny that aid bio-prospecting in the traditional medicinal plant genus Ferula L.(Apiaceae) in Iran.. J Med plants By-Prod..

[48] Salehi M, Naghavi MR, Bahmankar M (2019). A review of Ferula species: Biochemical characteristics, pharmaceutical and industrial applications, and suggestions for biotechnologists.. Ind Crops Prod..

[49] De Sarkar S, Sarkar D, Sarkar A, Dighal A, Chakrabarti S, Staniek K (2019). The leishmanicidal activity of artemisinin is mediated by cleavage of the endoperoxide bridge and mitochondrial dysfunction.. Parasitology..

[50] Ghodsian S, Taghipour N, Deravi N, Behniafar H, Lasjerdi Z (2020). Recent researches in effective antileishmanial herbal compounds: narrative review.. Parasitol Res..

[51] Gharirvand Eskandari E, Setorki M, Doudi M (2020). Medicinal Plants With Antileishmanial Properties: A Review Study.. Pharm Biomed Res..

[52] Islek Z, Ucisik MH, Sahin F (2022). Novel dual-fluorescent flow cytometric approach for quantification of macrophages infected with Leishmania infantum parasites.. Parasitology..

[53] Ilaghi M, Sharifi I, Sharififar F, Sharifi F, Oliaee RT, Babaei Z (2021). The potential role and apoptotic profile of three medicinal plant extracts on Leishmania tropica by MTT assay, macrophage model and flow cytometry analysis.. Parasite Epidemiol Control..

[54] Gharaei R, Akrami H, Heidari S, Asadi MH, Jalili A (2013). The suppression effect of Ferula gummosa Boiss. extracts on cell proliferation through apoptosis induction in gastric cancer cell line.. Eur J Integr Med..

[55] Mousavi SH, Davari AS, Iranshahi M, Sabouri-Rad S, Tayarani Najaran Z (2015). Comparative analysis of the cytotoxic effect of 7-prenyloxycoumarin compounds and herniarin on MCF-7 cell line.. Avicenna J Phytomed..

